# Video-Assisted versus Open Lobectomy in Patients with Compromised Lung Function: A Literature Review and Meta-Analysis

**DOI:** 10.1371/journal.pone.0124512

**Published:** 2015-07-06

**Authors:** Ruoyu Zhang, Mark K. Ferguson

**Affiliations:** 1 Division of Thoracic Surgery, Klinik Schillerhoehe, Center for Pneumology and Thoracic Surgery, Teaching Hospital of the University of Tuebingen, Gerlingen, Germany; 2 Department of Surgery and The Cancer Research Center, The University of Chicago, Chicago, Illinois, United States of America; University Hospital Oldenburg, GERMANY

## Abstract

**Background:**

It has been suggested that video-assisted (VATS) lobectomy is safer than open lobectomy in patients with compromised lung function, but data regarding this are limited. We assessed acute outcomes of VATS compared to open lobectomy in these high-risk patients using a systematic literature review and meta-analysis of data.

**Methods:**

The databases PubMed and Scopus were searched for studies published between 2000 and 2013 that reported mortality and morbidity of VATS in high-risk lung cancer patients defined as having compromised pulmonary or cardiopulmonary function. Study selection, data collection and critical assessment of the included studies were performed according to the recommendations of the Cochrane Collaboration.

**Results:**

Three case-control studies and three case series that included 330 VATS and 257 open patients were identified for inclusion. Operative mortality, overall morbidity and pulmonary morbidity were 2.5%, 39.3%, 26.2% in VATS patients and 7.8%, 57.5%, 45.5% in open lobectomy group, respectively. VATS lobectomy patients experienced significantly lower pulmonary morbidity (RR = 0.45; 95% CI, 0.30 to 0.67; *p* = 0.0001), somewhat reduced operative mortality (RR = 0.51; 95% CI, 0.24 to 1.06; *p* = 0.07), but no significant difference in overall morbidity (RR = 0.68; 95% CI, 0.41 to 1.14; *p* = 0.14).

**Conclusion:**

The existing data suggest that VATS lobectomy is associated with lower risk for pulmonary morbidity compared with open lobectomy in lung cancer patients with compromised lung function.

## Introduction

Up to 25% of patients with stage I non-small cell lung cancer (NSCLC) are considered ineligible for open lobectomy due to severe medical comorbidity [1]. Patients with compromised pulmonary function or cardiopulmonary reserve are considered high risk for postoperative complications and therefore more likely subjected to alternative treatment modalities, the outcomes of which are currently not as favorable as surgical resection [2–5]. Video-assisted (VATS) lobectomy has at least equal oncological efficacy and long-term outcomes in comparison to open lobectomy for early stage NSCLC [6–10]. Due to the theoretical advantage of preserved chest wall mechanics and the demonstrated advantage of less postoperative pain, VATS lobectomy is associated with lower postoperative morbidity compared to open lobectomy, and has been increasingly used since its introduction in early 1990s [5,11–15]. These benefits are believed to be greater for high-risk patients, defined as having compromised pulmonary function or cardiopulmonary reserve, and may broaden the applicability of curative lobectomy for this patient group [2,3,16].

To date, there is no published randomized controlled trial concerning VATS lobectomy specifically in NSCLC patients with compromised lung function. In large-scale retrospective studies reporting acute outcomes of VATS lobectomy, data for these high risk patients were not extractable [17–19]. The existing studies regarding this are limited by small sample size and variations in definitions and results, resulting in controversy over the clinical benefits of the minimally invasive approach compared to the open approach for this specific patient group [3,4,16,20–22]. We assessed the operative mortality and postoperative morbidity of VATS lobectomy for NSCLC patients with compromised lung function and compared them with open lobectomy using a systematic literature review and meta-analyses.

## Materials and Methods

Methods of the present systematic review and meta-analysis were specified in advance and documented in a protocol.

### Protocol for systematic review

A PICO-formatted matrix was developed to guide selection of appropriate search terms [23]. The population of interest (P) was physiologic high-risk NSCLC patients with compromised pulmonary function or cardiopulmonary reserve. Compromised pulmonary function was defined as predicted postoperative forced expiratory volume in the first second (FEV_1_) or diffusing capacity for carbon monoxide (DLCO) expressed as a percent predicted (ppoFEV_1_% or ppoDLCO%) ≤ 40. If ppoFEV_1_% or ppoDLCO% were not available, pulmonary function was considered compromised for preoperative FEV_1_% or DLCO% < 50 or FEV_1_ < 0.8 L. Compromised cardiopulmonary reserve was defined as peak oxygen consumption during exercise (VO_2_) < 40% predicted or < 12 mL·kg^-1^·min^-1^. The intervention (I) was VATS lobectomy. All studies reporting the mortality and morbidity following VATS lobectomy in patients with compromised pulmonary function or cardiopulmonary reserve were eligible for inclusion. The comparator (C) was similar patients undergoing open lobectomy. Primary outcomes (O) measured in the present systematic review were 1) operative mortality, defined as death during the hospitalization for lung resection or within 30 days of the operation; and 2) overall morbidity, defined as the occurrence of at least one major postoperative complication. The secondary outcomes were 1) pulmonary morbidity, defined as pneumonia, atelectasis requiring bronchoscopy, adult respiratory distress syndrome, air leak >5 days, initial ventilator support >24 hours, reintubation, and tracheostomy; and 2) cardiac morbidity, defined as acute myocardial infarction based on electrocardiographic or biochemical findings, congestive heart failure, and atrial or ventricular arrhythmia requiring intervention.

### Search strategy and study selection

PubMed and Scopus were searched for studies published in English between 2000 and 2013. The potentially eligible studies were selected by combining the search results regarding the population of interest, intervention, comparator and outcome (Table 1). In order to include studies on VATS lobectomy that included NSCLC patients as a subgroup, search terms such as NSCLC, lung cancer were not used. The authors had collected six citations in the past, which address the topic being posed in the present systematic review [3,4,16,20–22]. All of these citations were included in the search results, verifying the precision and the validity of the search strategy.

**Table 1:** Search terms.

Domain	Search terms	Boolean operator
Population of interest	Co-morbidity, co-morbidities, comorbidity, comorbidities, "pulmonary function", "lung function", "pulmonary function test", "pulmonary function tests", "lung function test", "lung function tests", "cardiopulmonary reserve".	OR
Intervention	Lobectomy, lobectomies, "lung resection", "lung resections", "pulmonary resection", "pulmonary resections", pneumonectomy^a^.	OR
Comparator	VATS, "minimally invasive thoracic surgery", "minimally invasive thoracic surgeries", "video-assisted thoracic surgery", "video-assisted thoracic surgeries", "video-assisted thoracoscopic surgery", "video-assisted thoracoscopic surgeries", " video-assisted thoracoscopic resection", "video-assisted thoracoscopic resections", thoracoscopic, endoscopic, "thoracic surgery, video-assisted", "minimally invasive surgery", "minimally invasive surgeries", "video-assisted surgery", "video-assisted surgeries", "video-assisted resection", "video-assisted resections", "minimally invasive resection", "minimally invasive resections".	OR
Outcome	Outcome, outcomes, complication, complications, treatment outcome	OR

^a^MeSH major topic, only for PubMed.

After discarding the duplicates, the records were further selected according to the inclusion and exclusion criteria (Table 2) that were defined a priori. In addition, potentially eligible studies were also identified by reading the review articles and other publications that emerged from the search. The studies arising from the search were reviewed and selected by the two authors independently. Disagreements were resolved by consensus.

**Table 2:** Criteria for considering studies.

Inclusion criteria	Exclusion criteria
The study reports the mortality and morbidity after VATS lobectomy for NSCLC regardless of stage	Review article
The study involves adult physiologic high risk patients	Duplicate report by the same institution
The technique of VATS lobectomy is consistent with the CALBG definition (anatomic lobectomy; individual ligation of hilar structures; 1 to 3 ports; no rib spreading; video monitor used for guidance; mediastinal/hilar nodal sampling or dissection)	Fewer than 10 VATS lobectomy cases in the study
The contribution of segmental resection or pneumonectomy cases is <10%	

VATS = video-assisted thoracic surgery, NSCLC = non-small cell lung cancer, CALGB = Cancer and Leukemia Group B.

### Data collection and critical assessment

In accordance with the recommendation of the Cochrane Collaboration, a data collection form was designed and tested [24]. Using the data collection form, the characteristics, intention, methods and outcomes of interest in each intervention group were assessed. The authors also extracted data of interest indirectly from information presented in the text and tables of full-text articles. Original authors were not contacted with a request to provide data that were not extractable from the publications.

Rather than being summarized in an overall score, the quality of each selected study was evaluated regarding selection bias, performance bias, detection bias, attrition bias and reporting bias according to the recommendation of the Cochrane Collaboration [25]. The risk of bias for each domain was judged as ‘low risk’, ‘high risk', or ‘unclear risk’. In the present systematic review, 'unclear risk' indicates either lack of information or uncertainty over the potential for bias.

### Statistical analysis

A meta-analysis was performed using Review Manager Version 5.0 (Cochrane Collaboration, Software Update, Oxford, United Kingdom). The risk ratio (RR) between VATS lobectomy and open lobectomy groups with 95% confidence intervals was used as a summary statistic for effect measures. The chi-squared test was used to assess heterogeneity between studies. I^2^ statistic was used to estimate the percentage of total variation across studies due to heterogeneity. I^2^ of more than 50% was considered substantial heterogeneity. A fixed-effects model was adopted if the level of heterogeneity was acceptable (*p* > 0.10, or *p* ≤ 0.10 but I^2^ ≤ 50%); otherwise, a random-effects model was adopted. Sensitivity analyses were performed to evaluate the robustness of the results of meta-analyses bases on study quality. When data were available, an intention-to-treat analysis was preferred to reduce the selection bias. Continuous data are presented as median and range. A *p*-value <0.05 was considered statistically significant.

## Results

### Description of studies

**Fig 1:**
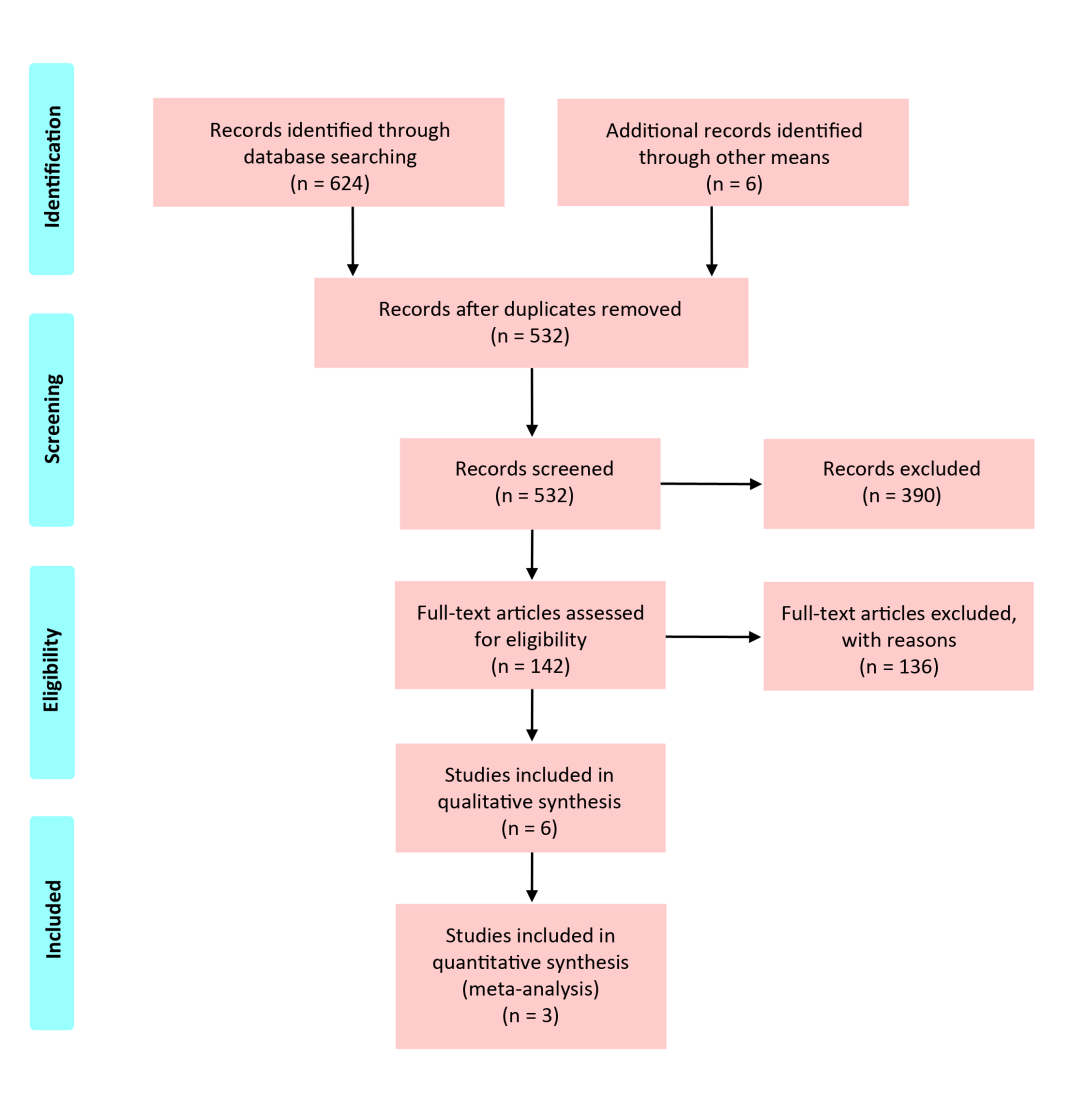
Diagram of literature selection.

A total of 368 and 256 publications were identified in PubMed and Scopus databases, respectively. After discarding duplicates, 526 publications were screened based on title and abstract. Of those, 142 publications were further assessed for eligibility based on full-text articles. Six publications ultimately were included for data collection and critical assessment [3,4,16,20,22,26]. A list identifying reasons for exclusion of the 136 publications is available as supporting information (S1 Table [https://drive.google.com/folderview?id=0B8uBM3vcuLv8d2p1cEFBc3NGZU0&usp=sharing]). No additional eligible publication was identified after assessing review articles. The diagram of literature selection is depicted in Fig 1. The publications were excluded mainly because they did not involve the population of interest (n = 316, 50.6%) or the clinical data of the population of interest were not extractable (n = 88, 14.1%).

The included publications consisted of three case-control studies and three case series. The characteristics of the studies are listed in Table 3. A total of 620 NSCLC patients with compromised pulmonary function were involved, of whom 330 underwent VATS lobectomy and 257 underwent open lobectomy. Most studies were published in the last four years [4,16,20,22,26]. In patients with initially attempted VATS lobectomy, the conversion rate was 4.4%, ranging from 0% to 33.3%. The proportion of patients with pathologic stage I NSCLC was 61.4%, ranging from 45.9% to 82.1%. In the study of Berry et al., data regarding pulmonary morbidity were extracted from the subgroup patients with FEV_1_% or DLCO% ≤ 45 as the population of interest [20]. The intervention group in the study of Lau and co-workers comprised 27 patients undergoing open segmentectomies by thoracotomy in addition to 18 patients undergoing VATS lobectomies [4].

**Table 3:** Characteristics of studies.

Study	Year of publica-tion	Design	Country	Total number of patients	Number of VATS patients	Definition of compromised lung function
Berry et al.^20^	2010	CCS	USA	340	173	FEV_1_% or DLCO% ≤ 60
Lau et al.^4^	2010	CCS	UK	84	18	ppoFEV_1_ <40
Kachare et al.^16^	2011	CCS	USA	60	47	ppoFEV_1_% or ppoDLCO%<40
Garzon et al.^3^	2006	CS	China	25	13	FEV_1_ <0.8L or FEV1% <50
Paul et al.^26^	2013	CS	USA	50	18	ppoDLCO% ≤40
Wang et al.^22^	2013	CS	China	61	61	FEV_1_% <50

CCS = case-control study, CS = case series, FEV_1_ = forced expiratory volume in the first second, DLCO = diffusing capacity of the lung for carbon monoxide, FEV1% = FEV_1_ as a percent predicted DLCO% = DLCO as a percent predicted, ppoFEV_1_% = predicted postoperative FEV_1_ expressed as a percent predicted, ppoDLCO% = predicted postoperative DLCO expressed as a percent predicted.

### Risk of bias assessment

All included case-control studies suffer from the inherent bias of retrospective trials. Table 4 shows judgments about each type of bias for the included studies. As no cohort study or randomized, controlled trial was included, the assessment of attrition bias was omitted. Regarding selection bias, the data in all studies were collected based on a retrospective review of a clinical database and/or medical charts in a single center. The allocation to VATS or open approaches depended mainly on clinical decision making, the preference of surgeons, and patients’ preferences.

**Table 4:** Risk of bias summary.

Study	Selection bias	Performance bias	Detection bias	Reporting bias
Berry et al.^20^	High	High	High	Low
Lau et al.^4^	High	High	High	High
Kachare et al.^16^	High	High	High	High
Garzon et al.^3^	High	High	High	High
Paul et al.^26^	High	High	High	High
Wang et al.^22^	High	High	High	High

All case-control studies demonstrated significant imbalance in baseline characteristics [4,16,20]. In the study of Berry et al., the patients undergoing VATS lobectomy were significantly older and had more congestive heart failure, but had less advanced stage NSCLC, less previous thoracic surgery, and less preoperative chemotherapy or radiation compared to those undergoing open lobectomy [20]. In the study of Kachare et al., the patients in VATS group were also older and there was no report on co-morbidities [16]. In the study of Lau et al., the patients in the VATS group had more early-stage NSCLC, and there was no report on cardiac co-morbidities and previous malignancies [4]. Only the study of Kachare et al. presented the results of intention-to-treat analysis [16].

### Operative mortality

**Fig 2:**
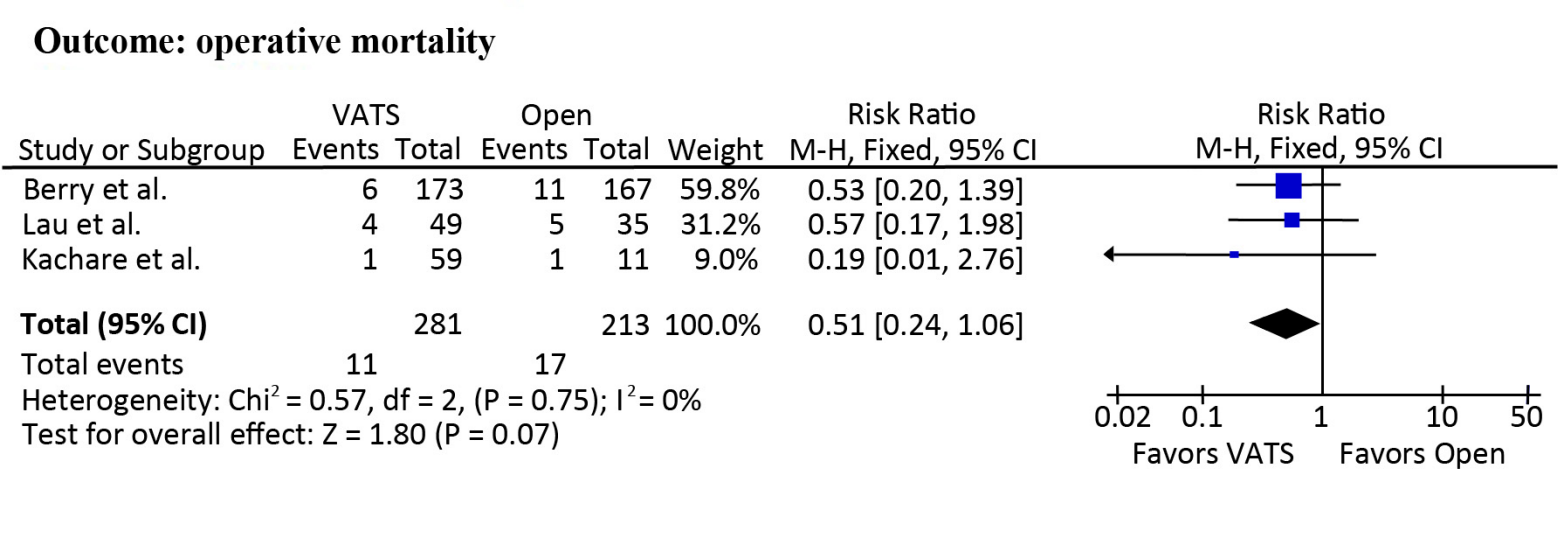
Meta-analyses of operative mortality.

Operative mortality was 2.5% (0% to 8.2%) and 7.8% (0% to 14.3%) in patients undergoing VATS lobectomy and open lobectomy, respectively. Meta-analyses demonstrated a trend towards reduced risk of operative mortality in patients undergoing VATS lobectomy (RR = 0.51; 95% CI 0.24 to 1.06; *p* = 0.07; Fig 2). The publication of Paul et al. was not included in this meta-analysis because there was no operative mortality in either the intervention or control groups [26]. In addition, the VATS group (n = 49) in the study of Lau et al. included 27 patients undergoing open segmentectomy [4]. The sensitivity analysis, from which the study of Lau et al. was removed, did not show a difference between the two approaches (RR = 0.48; 95% CI, 0.19 to 1.20; *p* = 0.12).

### Overall morbidity

**Fig 3:**
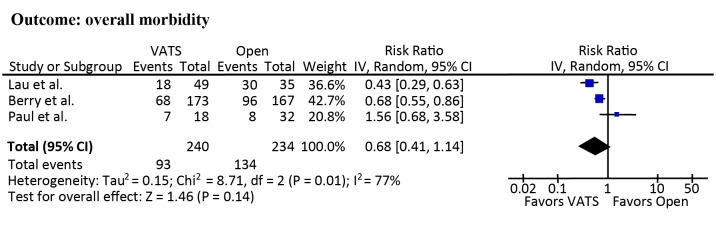
Meta-analyses of overall morbidity.

Other than the study of Kachare et al., data on overall morbidity could be extracted directly and indirectly from the included studies [16]. The overall morbidity was 39.3% (36.7% to 46.2%) and 57.5% (25.0% to 85.7%) in patients undergoing VATS lobectomy and open lobectomy, respectively. The overall morbidity in the study of Kachare et al. could not be accurately calculated because only the incidence of individual postoperative complications was reported [16]. Meta-analyses demonstrated the risk of overall morbidity between VATS and open lobectomy was comparable (RR = 0.68; 95% CI, 0.41 to 1.14; *p* = 0.14; Fig 3).

### Pulmonary morbidity

**Fig 4:**
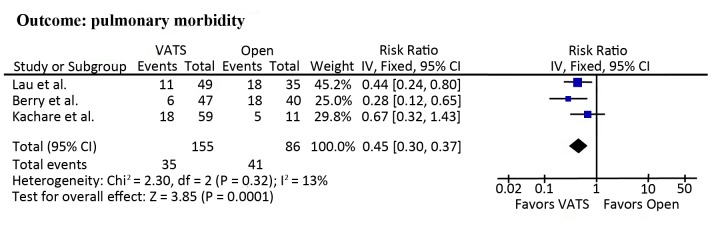
Meta-analyses of pulmonary morbidity.

All included studies reported pulmonary morbidity, which was 26.2% (12.8% to 36.1%) and 45.5% (45.0% to 51.4%) in patients undergoing VATS lobectomy and open lobectomy, respectively. Meta-analyses demonstrated a significantly lower risk of pulmonary morbidity in patients undergoing VATS lobectomy compared to those undergoing open lobectomy (RR = 0.45; 95% CI, 0.30 to 0.67; *p* = 0.0001; Fig 4). Sensitivity analysis was performed due to absence of a clear definition of pulmonary morbidity in the study of Lau et al. [4]. However, the results remained highly significant (RR = 0.45; 95% CI, 0.26 to 0.79; *p* = 0.005).

### Cardiac morbidity

Only one case-control study and two case series reported cardiac morbidity [16,21,22]. While no myocardial infarction occurred in the two case series, the incidence of myocardial infarction was 1.7% and 9.1% in patients undergoing VATS lobectomy and open lobectomy, respectively, in the case-control study of Kachare et al. [16]. The incidence of atrial fibrillation was 9.0% (4.9% to 12.0%) and 8.6% (0% to 17.1%) in patients undergoing VATS lobectomy and open lobectomy, respectively. A meta-analysis was not performed because comparisons were only available in two studies.

## Discussion

Despite the advance in the non-surgical therapy modalities, lobectomy remains the therapy of choice for early-stage lung cancer due to the favorable oncological outcomes and survival rates [12,27]. In the last decade VATS lobectomy has been increasingly adopted for treatment of early-stage NSCLC and has achieved at least equal oncological efficacy and long-term outcomes in comparison to open lobectomy [6–10]. An ample body of research demonstrates that VATS lobectomy is associated with better preservation of lung function in the initial postoperative period and fewer pulmonary complications [5,11,14–16,22,28]. On the other hand, a substantial proportion of early-stage lung cancer patients is of questionable eligibility for open lobectomy according to conventional risk assessment due to compromised pulmonary function or cardiopulmonary reserve [1–5]. In this context, the clinical benefits of VATS lobectomy may permit a safe anatomic resection in high-risk NSCLC patients with compromised pulmonary function or cardiopulmonary reserve, who traditionally would have been offered non-surgical therapy [29,30]. However, there is insufficient information about the potential advantages of VATS lobectomy for this specific patient group.

In the present systematic review we assessed the operative mortality and postoperative morbidity of VATS lobectomy for high-risk NSCLC patients based on the available published data. A total of three case-control studies and three case series published between 2000 and 2013 were included after searching the most comprehensive medical databases. A multitude of randomized clinical trials and large-scale retrospective studies on VATS lobectomy in NSCLC patients exist for this period [7,18,19,31–33]. However, high risk patients were either not involved, or the data for this special patient group were not extractable. In a case-control study based on the STS General Thoracic Database, Ceppa et al. analyzed the clinical data of 12,970 patients who underwent an anatomic pulmonary resection (either lobectomy or segmentectomy) by either thoracotomy (n = 8439) or VATS (n = 4531) between 2000 and 2010 [17]. They found that thoracotomy was associated with markedly increased pulmonary complications in patients with impaired pulmonary function when compared with VATS patients. Besides the significant baseline imbalances as one of the major limitations, the exact number of patients with impaired pulmonary function, which was defined as predicted FEV_1_% < 60, was not extractable in this study.

As one of our major findings, the risk for pulmonary morbidity after VATS lobectomy in high-risk patients was less than half of that in their counterparts undergoing open lobectomy. In the last decade, a number of randomized clinical trials and large propensity-matched studies have shown that VATS lobectomy for early-stage NSCLC resulted in lower pulmonary morbidity compared to the open approach [7,18,19,31–33]. The reasons behind these advantages may be related to less chest wall trauma, reduced acute postoperative pain and earlier removal of chest tube, resulting in better preservation and faster recovery of pulmonary function after VATS lobectomy [5,11,14,15,28]. Indeed, better preserved pulmonary function in the immediate postoperative phase has been found to be strongly associated with lower pulmonary morbidity after lobectomy [34]. Moreover, VATS lobectomy enables improved deep breathing, early expectoration and ambulation in the postoperative period, which contribute to a decreased risk for pulmonary morbidity [16,22].

Due to advances in perioperative care in the last decade, operative mortality in high-risk patients has decreased after VATS and open lobectomy. Our meta-analyses did not demonstrate advantages of VATS lobectomy in high-risk patients in this regard. This finding might be explained by the limited patient numbers involved in the present systematic review and meta-analysis, and is similar to findings previously reported in a meta-analysis of outcomes for lobectomy patients in all risk levels [35].

Currently, non-surgical treatments including stereotactic body radiotherapy and radiofrequency ablation are increasingly employed in high-risk NSCLC patients due to low procedural mortality and morbidity [12,36]. However, accumulating evidence indicates that these treatment modalities are associated with increased risk for involved lobe and regional recurrence compared with lobectomy [1]. In contrast, concerns regarding the oncologic efficacy and long-term outcome of VATS lobectomy for early-stage NSCLC have not been supported by evidence in the recent literature [10,37,38]. In a prospective randomized clinical trial a total of 100 patients with clinical stage IA NSCLC underwent either VATS lobectomy or open lobectomy [37]. The 3- and 5-year survival rates as well as incidence of recurrences of VATS group were found equivalent to that of open group. Recently Su and colleagues performed a secondary analysis of clinical data in 1,018 patients enrolled in a large-scale multicenter, randomized trial (ACOSOG Z0030), which was conducted to determine the long-term clinical outcomes of patients undergoing surgical treatment for early stage NSCLC [10]. They found no difference in overall survival, disease-free survival, survival based on pattern of recurrence or time to locoregional recurrence between the VATS and open lobectomy groups during a median follow-up period of 6.7 years.

The authors acknowledge the limitations of the present systematic review. Besides the inherent bias of the retrospective studies and relatively small number of involved patients, the included case-control studies demonstrated significant imbalances in baseline characteristics. Also of concern is the fact that the definition of compromised pulmonary function varied in the included studies, resulting in increased heterogeneity of the population of interest. In addition, intention-to-treat analysis of the acute outcome of VATS lobectomy was reported only in one out of six included studies [16]. In the remaining studies, patients intended for VATS lobectomy but converted to open thoracotomy were included in the open lobectomy group, resulting in reduced validity of the pooled outcomes and increased bias in favor of VATS lobectomy [7,39]. Despite the imperfect data of the included studies, the results of the present systematic review and meta-analysis are intriguing and suggestive of a potential benefit of VATS lobectomy in high-risk patients.

## Conclusions

The existing clinical studies suggest that VATS lobectomy is associated with a lower incidence of pulmonary morbidity compared to open lobectomy in high risk patients. Our investigation encourages careful consideration of VATS lobectomy for early-stage NSCLC in high-risk patients. Prospective comparative trials are warranted for further evaluation of the potential clinical benefits of VATS lobectomy in this patient population.

## Supporting Information

S1 Diagram Flow diagram of literature selection. (DOC)

S1 Protocol Protocol for the present systematic review and meta-analysis. (DOCX)

S1 Table Articles excluded from meta-analysis of VATS vs open lobectomy in patients with compromised pulmonary function. (DOCX)

S2 Table PRISMA checklist. (DOC)
