# Supplementary material for: Video-Assisted versus Open Lobectomy in Patients with Compromised Lung Function: A Literature Review and Meta-Analysis
Source: PLoS One. 2015 Jul 6;10(7):e0124512. doi: 10.1371/journal.pone.0124512 (PMC4493021; doi:10.1371/journal.pone.0124512)
Supplement: S1 Diagram — (DOC) [file pone.0124512.s001.doc]

**Screening**

**Included**

**Eligibility**

**Identification**

Records identified through database searching
(n = 624 )

Additional records identified through other sources
(n = 6 )

Records after duplicates removed
(n = 532 )

Records screened
(n = 532 )

Records excluded
(n = 390 )

Full-text articles assessed for eligibility
(n = 142 )

Full-text articles excluded, with reasons
(n = 136 )

Studies included in qualitative synthesis
(n = 6 )

Studies included in quantitative synthesis (meta-analysis)
(n = 3 )
