# Supplementary material for: Video-Assisted versus Open Lobectomy in Patients with Compromised Lung Function: A Literature Review and Meta-Analysis
Source: PLoS One. 2015 Jul 6;10(7):e0124512. doi: 10.1371/journal.pone.0124512 (PMC4493021; doi:10.1371/journal.pone.0124512)
Supplement: S1 Protocol — (DOCX) [file pone.0124512.s002.docx]

# Protocol of The Systematic Review and Meta-Analysis on

# Video-assisted Versus Open Lobectomy in High Risk Patients

Ruoyu Zhang^1^, Mark K. Ferguson^2^

^1^ Section of Thoracic, Cardiac, Transplant and Vascular Surgery, Department of Surgery, The Hannover Medical School, Hannover, Germany

^2^ Section of Cardiac and Thoracic Surgery, Department of Surgery, The University of Chicago, Chicago, USA

#

# Background

## Description of the condition

Up to 25% of patients with stage I NSCLC are not eligible for open lobectomy due to severe medical comorbidity. This patient group is more likely subjected to alternative treatment modalities, whose outcome is currenctly not as favorable as radical surgical resection.

## Description of the intervention

To date, VATS lobectomy (VATSL) has been accepted as a curative, but less invasive therapy for early-stage NSCLC and an alternative to open lobectomy with at least equal oncological efficacy and long-term outcome.

## How the intervention might work

Due to its minimally invasive nature, VATSL is associated with less postoperative pain and preservation of chest wall mechanics, resulting in reduced postoperative morbidity compared with open lobectomy by thoracotomy.

## Why it is important to do this review

Controversy still exists regarding the safety of this approach in high risk patients. Prospective randomized trails concerning this specific topic are lacking. The published case-control studies and case series vary in sample size, definitions and results.

# Objectives

To assess the safety of VATSL for NSCLC in high risk patients by literature review, and to compare them with open lobectomy by meta-analysis.

#

# Methods

## Criteria for considering studies for this review

### Types of studies

All studies will be considered eligible if they meet the following criteria.

The inclusion criteria are:

1. it reports the postoperative mortality and morbidity after VATSL for NSCLC (regardless stages),
2. the percentage of other VATS anatomic resections (the combination of segmentectomies and pneumonectomies) is less than 10%,
3. it involves adult physiologic high risk patients [see below for definition], at least as a subgroup;
4. the technique of VATSL is consist to the definition of CALGB (anatomic lobectomy with individual ligation of lobar vessels and bronchus, as well as hilar and mediastinal lymph node dissection or sampling, using the video screen for guidance, one, two or three ports, and no retractor use or rib spreading),
5. it is published between January 1st. 2000 and December 31st 2013.

The exclusion criteria are:

1. review articles,

2. the one of best quality will be used in repeated reports by the same institution,

3. the cases of VATSL in the study are less than 10.

### Types of participants

The population of interest is physiologic high risk NSCLC patients with compromised pulmonary function or cardiopulmonary reserve.

Compromised pulmonary function is defined as predicted postoperative FEV1 or DLCO expressed as a percent predicted (ppoFEV_1_% or ppoDLCO%) ≤ 40. If ppoFEV1% or ppoDLCO% are not available, pulmonary function is considered compromised if preoperative FEV_1_% or DLCO% < 50 or FEV_1_ < 0.8 L.

Compromised cardiopulmonary reserve was defined as peak VO_2_ < 40% predicted or < 12 mL•kg^-1^•min^-1^ [1-4].

### Types of interventions

VATSL with or without comparison to open lobectomy by thoracotomy.

All studies reporting the postoperative mortality and morbidity after VATSL in high risk patients are eligible for literature review. However, only studies comparing VATSL and open lobectomy by thoracotomy in this specific patient population will be included for meta-analysis.

### Types of outcome measures

#### Primary outcomes are:

#### operative mortality, defined as death during the hospitalization for lung resection or within 30 days of the operation,

#### overall morbidity, defined as the occurrence of at least one major postoperative complication.

#### Secondary outcomes are:

#### pulmonary morbidity, defined as those occurred during hospitalization for lung resection or within 30 days of surgery and include pneumonia, atelectasis requiring bronchoscopy, adult respiratory distress syndrome, air leak >5 days, initial ventilator support >24 hours, reintubation, tracheostomy;

#### cardiac morbidity, defined as those occurred during hospitalization for lung resection or within 30 days of surgery and include acute myocardial infarction based on electrocardiograhic or biochemical findings, congestive heart failure, atrial or ventricular arrhythmia requiring intervention.

## Search methods for identification of studies

The databases PubMed and Scopus will be searched for eligible studies. The search terms are listed in Table 1 in Appendix. In addition, eligible studies will also be identified from review articles.

## Data collection and analysis

### Selection of studies

The identified studies will be reviewed and selected by two authors independently. Disagreements between reviewers will be resolved by consensus.

### Data extraction and management

The data will be extracted from eligible studies by two investigators independently.

### Assessment of risk of bias in included studies

The risk of bias in included studies will be evaluated with the Cochrane Collaboration’s tool for assessing risk of bias by two investigators independently. The Disprepancies will be resolved by consensus.

### Measures of treatment effect

Risk ratio (RR) between VATSL group and open lobectomy group with 95% confidence intervals will be used as a summary statistic for effect measures.

### Assessment of heterogeneity

The chi-squared test will be used to assess heterogeneity between trials. I^2^ statistic will be used to estimate the percentage of total variation across studies due to heterogeneity. I^2^ of more than 50% will be considered substantial heterogeneity. In case of a substantial heterogeneity, the possible clinical and methodological diversity will be explored qualitatively.

### Assessment of reporting biases

Publication bias will be explored through visual inspection of the funnel plots.

### Data synthesis

Fixed-effects models will be adopted if the level of heterogeneity is acceptable (*p* > 0.10, or *p* ≤ 0.10 but I^2^ ≤ 50%); otherwise, random-effects model will be adopted.

**Appendix:**

Table 1: search terms.

| **Domain** | **Search terms** | **Boolean operator** |
| --- | --- | --- |
| Population of interest | Co-morbidity, co-morbidities, comorbidity, comorbidities, "pulmonary function", "lung function", "pulmonary function test", "pulmonary function tests", "lung function test", "lung function tests", "cardiopulmonary reserve". | OR |
| Intervention | Lobectomy, lobectomies, "lung resection", "lung resections", "pulmonary resection", "pulmonary resections", pneumonectomy^a^. | OR |
| Comparator | VATS, "minimally invasive thoracic surgery", "minimally invasive thoracic surgeries", "video-assisted thoracic surgery", "video-assisted thoracic surgeries", "video-assisted thoracoscopic surgery", "video-assisted thoracoscopic surgeries", " video-assisted thoracoscopic resection", "video-assisted thoracoscopic resections", thoracoscopic, endoscopic, "Thoracic Surgery, Video-Assisted"*, "minimally invasive surgery", "minimally invasive surgeries", "video-assisted surgery", "video-assisted surgeries", "video-assisted resection", "video-assisted resections", "minimally invasive resection", "minimally invasive resections". | OR |
| Outcome | Outcome, outcomes, complication, complications, Treatment Outcome* | OR |

^a^MeSH major topic, only for PubMed

**References**

1. Demmy TL, Curtis JJ. Minimally invasive lobectomy directed toward frail and high-risk patients: a case-control study. The Annals of thoracic surgery. 1999;68(1):194-200.
2. Ceppa DP, Kosinski AS, Berry MF, Tong BC, Harpole DH, Mitchell JD, et al. Thoracoscopic lobectomy has increasing benefit in patients with poor pulmonary function: a Society of Thoracic Surgeons Database analysis. Annals of surgery. 2012;256(3):487-93.
3. Poonyagariyagorn H, Mazzone PJ. Lung cancer: preoperative pulmonary evaluation of the lung resection candidate. Semin Respir Crit Care Med. 2008 Jun;29(3):271-84.
4. von Groote-Bidlingmaier F, Koegelenberg CF, Bolliger CT. Functional evaluation before lung resection. Clin Chest Med. 2011 Dec;32(4):773-82.
