# Supplementary material for: Video-Assisted versus Open Lobectomy in Patients with Compromised Lung Function: A Literature Review and Meta-Analysis
Source: PLoS One. 2015 Jul 6;10(7):e0124512. doi: 10.1371/journal.pone.0124512 (PMC4493021; doi:10.1371/journal.pone.0124512)
Supplement: S1 Table — (DOCX) [file pone.0124512.s003.docx]

S1 Table. Articles excluded from meta-analysis of VATS vs open lobectomy in patients with compromised pulmonary function.

Ruoyu Zhang, MD

Mark K. Ferguson, MD

A total of 136 publications were excluded based on full-text review. They included 38 studies not involving the population of interest [1-38], 87 studies with inextractable clinical data of the population of interest [39-125], five studies, in which the VATS lobectomy technique was not in accordance with the definition of CALGB [126-130], one study with small sample size [131], two publications merely describing operative techniques [132,133] and two duplicates [3,68] as well as one review article [134].

*References:*

1. Benhamed L, Bellier J, Fournier C, Akkad R, Mathieu D, et al. (2011) Postoperative ischemic bronchitis after lymph node dissection and primary lung cancer resection. Ann Thorac Surg 91: 355-359.

2. Deslee G, Barbe C, Bourdin A, Durand-Zaleski I, Dutau H, et al. (2012) [Cost-effectiveness of lung volume reduction coil treatment in emphysema. STIC REVOLENS]. Rev Mal Respir 29: 1157-1164.

3. Endoh H, Yamamoto R, Satoh Y, Kuwano H, Nishizawa N (2013) Risk analysis of pulmonary resection for elderly patients with lung cancer. Surgery today 43: 514-520.

4. Guan L, Hong WJ, Hong ZP, Huang T (2013) Effect of high-altitude exposure on safety of complete video-assisted thoracoscopic surgery lobectomy in elderly patients with non-small cell lung cancer and analysis of prognosis. Tumor 33: 327-333.

5. Hansen HJ, Petersen RH, Christensen M (2011) Video-assisted thoracoscopic surgery (VATS) lobectomy using a standardized anterior approach. Surgical Endoscopy and Other Interventional Techniques 25: 1263-1269.

6. Hill SE, Keller RA, Stafford-Smith M, Grichnik K, White WD, et al. (2006) Efficacy of single-dose, multilevel paravertebral nerve blockade for analgesia after thoracoscopic procedures. Anesthesiology 104: 1047-1053.

7. Imperatori A, Rovera F, Rotolo N, Nardecchia E, Conti V, et al. (2006) Prospective study of infection risk factors in 988 lung resections. Surgical Infections 7: S-57-S-60.

8. Iwasaki A, Hamanaka W, Hamada T, Hiratsuka M, Yamamoto S, et al. (2007) Comparison between a case-matched analysis of left upper lobe trisegmentectomy and left upper lobectomy for small size lung cancer located in the upper division. Thoracic and Cardiovascular Surgeon 55: 454-457.

9. Jeon JH, Kang CH, Kim HS, Seong YW, Park IK, et al. (2013) Video-assisted thoracoscopic lobectomy in non-small-cell lung cancer patients with chronic obstructive pulmonary disease is associated with lower pulmonary complications than open lobectomy: a propensity score-matched analysis. Eur J Cardiothorac Surg.

10. Klena JW, Saari AF, Peterson DO, Collins C, Johnson JA (2003) Combined video-assisted thoracoscopic lung volume reduction surgery and lobectomy in a high-risk patient. Ann Thorac Surg 76: 2079-2080.

11. Koizumi K, Haraguchi S, Hirata T, Hirai K, Mikami I, et al. (2002) Video-Assisted Lobectomy in Elderly Lung Cancer Patients. Japanese Journal of Thoracic and Cardiovascular Surgery 50: 15-22.

12. Lin J, Iannettoni MD (2005) Fast-tracking: Eliminating roadblocks to successful early discharge. Thoracic Surgery Clinics 15: 221-228.

13. Mikami I, Koizumi K, Tanaka S (2001) Changes in right ventricular performance in elderly patients who underwent lobectomy using video-assisted thoracic surgery for primary lung cancer. Jpn J Thorac Cardiovasc Surg 49: 153-159.

14. Morano MT, Araujo AS, Nascimento FB, da Silva GF, Mesquita R, et al. (2013) Preoperative pulmonary rehabilitation versus chest physical therapy in patients undergoing lung cancer resection: a pilot randomized controlled trial. Arch Phys Med Rehabil 94: 53-58.

15. Morio A, Miyamoto H, Yamazaki A, Anami Y, Oh S, et al. (2000) [A case of primary lung cancer complicated with post-operative intractable pulmonary fistula]. Kyobu Geka 53: 1144-1147.

16. Mueller DK, Foiles SR (2007) Right mainstem bronchial kink after right upper lobectomy. Ann Thorac Surg 84: 1401.

17. Nomori H, Ohtsuka T, Horio H, Naruke T, Suemasu K (2003) Difference in the impairment of vital capacity and 6-minute walking after a lobectomy performed by thoracoscopic surgery, an anterior limited thoracotomy, an anteroaxillary thoracotomy, and a posterolateral thoracotomy. Surgery Today 33: 7-12.

18. Park JS, Kim HK, Choi YS, Kim J, Shim YM, et al. (2011) Unplanned conversion to thoracotomy during video-assisted thoracic surgery lobectomy does not compromise the surgical outcome. World Journal of Surgery 35: 590-595.

19. Paull DE, Thomas ML, Meade GE, Updyke GM, Arocho MA, et al. (2006) Determinants of quality of life in patients following pulmonary resection for lung cancer. Am J Surg 192: 565-571.

20. Rovera F, Imperatori A, Militello P, Morri A, Antonini C, et al. (2003) Infections in 346 consecutive video-assisted thoracoscopic procedures. Surgical Infections 4: 45-51.

21. Shapiro M, Weiser TS, Wisnivesky JP, Chin C, Arustamyan M, et al. (2009) Thoracoscopic segmentectomy compares favorably with thoracoscopic lobectomy for patients with small stage I lung cancer. Journal of Thoracic and Cardiovascular Surgery 137: 1388-1393.

22. Solaini L, Bagioni P, Prusciano F, Di Francesco F, Poddie DB (2000) Video-assisted thoracic surgery (VATS) lobectomy for typical bronchopulmonary carcinoid tumors. Surg Endosc 14: 1142-1145.

23. Ueda K, Tanaka T, Jinbo M, Yagi T, Li TS, et al. (2007) Sutureless pneumostasis using polyglycolic acid mesh as artificial pleura during video-assisted major pulmonary resection. Ann Thorac Surg 84: 1858-1861.

24. Uramoto H, Nakanishi R, Fujino Y, Imoto H, Takenoyama M, et al. (2001) Prediction of pulmonary complications after a lobectomy in patients with non-small cell lung cancer. Thorax 56: 59-61.

25. Varela G, Brunelli A, Rocco G, Jimenez MF, Salati M, et al. (2007) Evidence of lower alteration of expiratory volume in patients with airflow limitation in the immediate period after lobectomy. Ann Thorac Surg 84: 417-422.

26. Varela G, Brunelli A, Rocco G, Marasco R, Jimenez MF, et al. (2006) Predicted versus observed FEV1 in the immediate postoperative period after pulmonary lobectomy. Eur J Cardiothorac Surg 30: 644-648.

27. Varela G, Brunelli A, Rocco G, Novoa N, Refai M, et al. (2007) Measured FEV1 in the first postoperative day, and not ppoFEV1, is the best predictor of cardio-respiratory morbidity after lung resection. Eur J Cardiothorac Surg 31: 518-521.

28. Wang BY, Liu CC, Shih CS (2010) Short-term results of thoracoscopic lobectomy and segmentectomy for lung cancer in Koo foundation sun yat-sen cancer center. Journal of Thoracic Disease 2: 64-70.

29. Wislez M, Massiani MA, Milleron B, Souidi A, Carette MF, et al. (2003) Clinical characteristics of pneumonic-type adenocarcinoma of the lung. Chest 123: 1868-1877.

30. Witte B, Wolf M, Hillebrand H, Huertgen M (2013) Split-lobe resections versus lobectomy for lung carcinoma of the left upper lobe: a pair-matched case-control study of clinical and oncological outcomes. Eur J Cardiothorac Surg.

31. Yamamoto R, Tada H, Kishi A, Tojo T (2000) Effects of preoperative chemotherapy and radiation therapy on human bronchial blood flow. J Thorac Cardiovasc Surg 119: 939-945.

32. Yamashita JI, Kurusu Y, Fujino N, Saisyoji T, Ogawa M (2000) Detection of circulating tumor cells in patients with non-small cell lung cancer undergoing lobectomy by video-assisted thoracic surgery: a potential hazard for intraoperative hematogenous tumor cell dissemination. J Thorac Cardiovasc Surg 119: 899-905.

33. Yamauchi Y, Isaka M, Maniwa T, Takahashi S, Kurai H, et al. (2013) Chest tube tip culture as a predictor of postoperative infection in lung cancer operations. Annals of Thoracic Surgery 96: 1796-1802.

34. Yang CF, D'Amico TA (2012) Thoracoscopic segmentectomy for lung cancer. Ann Thorac Surg 94: 668-681.

35. Yang X, Wang S, Qu J (2009) Video-assisted thoracic surgery (VATS) compares favorably with thoracotomy for the treatment of lung cancer: a five-year outcome comparison. World J Surg 33: 1857-1861.

36. Zhong C, Fang W, Mao T, Yao F, Chen W, et al. (2012) Comparison of thoracoscopic segmentectomy and thoracoscopic lobectomy for small-sized stage IA lung cancer. Annals of Thoracic Surgery 94: 362-367.

37. Zhong C, Yao F, Zhao H (2013) Clinical outcomes of thoracoscopic lobectomy for patients with clinical N0 and pathologic N2 non-small cell lung cancer. Annals of Thoracic Surgery 95: 987-992.

38. Zurauskas A, Tikuisis R, Miliauskas P (2002) [Stair climbing test in prediction of postoperative complications after lung cancer surgery]. Medicina (Kaunas) 38 Suppl 2: 37-39.

39. Allen MS, Darling GE, Pechet TT, Mitchell JD, Herndon JE, 2nd, et al. (2006) Morbidity and mortality of major pulmonary resections in patients with early-stage lung cancer: initial results of the randomized, prospective ACOSOG Z0030 trial. Ann Thorac Surg 81: 1013-1019; discussion 1019-1020.

40. Amar D, Zhang H, Shi W, Downey RJ, Bains MS, et al. (2012) Brain natriuretic peptide and risk of atrial fibrillation after thoracic surgery. J Thorac Cardiovasc Surg 144: 1249-1253.

41. Amer K, Khan AZ, Vohra HA (2011) Video-assisted thoracic surgery of major pulmonary resections for lung cancer: The Southampton experience. European Journal of Cardio-thoracic Surgery 39: 173-179.

42. Augustin F, Schmid T, Lucciarini P, Bieck S, Bodner J (2010) Minimally invasive lung lobectomy: Indication, patient selection, surgical technique and outcome. European Surgery - Acta Chirurgica Austriaca 42: 204-208.

43. Balduyck B, Hendriks J, Lauwers P, Van Schil P (2007) Quality of life evolution after lung cancer surgery: a prospective study in 100 patients. Lung Cancer 56: 423-431.

44. Berry MF, Onaitis MW, Tong BC, Harpole DH, D'Amico TA (2011) A model for morbidity after lung resection in octogenarians. Eur J Cardiothorac Surg 39: 989-994.

45. Boffa DJ, Allen MS, Grab JD, Gaissert HA, Harpole DH, et al. (2008) Data from The Society of Thoracic Surgeons General Thoracic Surgery database: the surgical management of primary lung tumors. J Thorac Cardiovasc Surg 135: 247-254.

46. Bollen EC, Belgers E, van Haren EH, Siebenga J (2008) [Surgical treatment of lung cancer with complete VATS lobectomy, new in the Netherlands]. Ned Tijdschr Geneeskd 152: 1204-1209.

47. Cattaneo SM, Park BJ, Wilton AS, Seshan VE, Bains MS, et al. (2008) Use of Video-Assisted Thoracic Surgery for Lobectomy in the Elderly Results in Fewer Complications. Annals of Thoracic Surgery 85: 231-236.

48. Ceppa DP, Kosinski AS, Berry MF, Tong BC, Harpole DH, et al. (2012) Thoracoscopic lobectomy has increasing benefit in patients with poor pulmonary function: A society of thoracic surgeons database analysis. Annals of Surgery 256: 487-493.

49. Cui F, Liu J, Shao W, He J (2013) Thoracoscopic minimally invasive surgery for non-small cell lung cancer in patients with chronic obstructive pulmonary disease. J Thorac Dis 5: S260-266.

50. D'Amico TA, Niland J, Mamet R, Zornosa C, Dexter EU, et al. (2011) Efficacy of mediastinal lymph node dissection during lobectomy for lung cancer by thoracoscopy and thoracotomy. Annals of Thoracic Surgery 92: 226-232.

51. Dearmond DT, Simmons JD, Cline AM, Zarzabal LA, Johnson SB, et al. (2012) Feasibility of video-assisted thoracoscopic surgery lobectomy in Veterans Administration patients. American Journal of Surgery 204: e15-e20.

52. Duann CW, Hung JJ, Hsu PK, Huang CS, Hsieh CC, et al. (2013) Surgical outcomes in lung cancer presenting as ground-glass opacities of 3cm or less: A review of 5 years' experience. Journal of the Chinese Medical Association 76: 693-697.

53. Flores RM, Park BJ, Dycoco J, Aronova A, Hirth Y, et al. (2009) Lobectomy by video-assisted thoracic surgery (VATS) versus thoracotomy for lung cancer. Journal of Thoracic and Cardiovascular Surgery 138: 11-18.

54. Gonzalez D, de la Torre M, Paradela M, Fernandez R, Delgado M, et al. (2011) Video-assisted thoracic surgery lobectomy: 3-year initial experience with 200 cases. European Journal of Cardio-thoracic Surgery 40: e21-e28.

55. Gopaldas RR, Bakaeen FG, Dao TK, Walsh GL, Swisher SG, et al. (2010) Video-Assisted Thoracoscopic Versus Open Thoracotomy Lobectomy in a Cohort of 13,619 Patients. Annals of Thoracic Surgery 89: 1563-1570.

56. Hagan ME, Williams ST, Socci L, Malik M, Internullo E, et al. (2013) Completing the audit cycle improves surgical standards in lung cancer: Why do some patients still not receive the best care? Journal of Thoracic Oncology 8: 779-782.

57. Handy JR, Jr., Asaph JW, Douville EC, Ott GY, Grunkemeier GL, et al. (2010) Does video-assisted thoracoscopic lobectomy for lung cancer provide improved functional outcomes compared with open lobectomy? Eur J Cardiothorac Surg 37: 451-455.

58. Haraguchi S, Koizumi K, Hatori N, Hioki M, Yamashita K, et al. (2004) Postoperative Respiratory Complications of Video-assisted Thoracic Surgery for Lung Cancer. Journal of Nippon Medical School 71: 30-34.

59. Haruki T, Nakamura H, Taniguchi Y, Miwa K, Adachi Y, et al. (2010) 'Lung age' predicts post-operative complications and survival in lung cancer patients. Respirology 15: 495-500.

60. Hoksch B, Ablassmaier B, Walter M, Muller JM (2003) [Complication rate after thoracoscopic and conventional lobectomy]. Zentralbl Chir 128: 106-110.

61. Igai H, Takahashi M, Ohata K, Yamashina A, Matsuoka T, et al. (2009) Surgical treatment for non-small cell lung cancer in octogenarians--the usefulness of video-assisted thoracic surgery. Interact Cardiovasc Thorac Surg 9: 274-277.

62. Ilonen IK, Rasanen JV, Knuuttila A, Salo JA, Sihvo EI (2011) Anatomic thoracoscopic lung resection for non-small cell lung cancer in stage I is associated with less morbidity and shorter hospitalization than thoracotomy. Acta Oncol 50: 1126-1132.

63. Ishikawa S, Griesdale DE, Lohser J (2012) Acute kidney injury after lung resection surgery: incidence and perioperative risk factors. Anesth Analg 114: 1256-1262.

64. Jiang G, Yang F, Li X, Liu J, Li J, et al. (2011) Video-assisted thoracoscopic surgery is more favorable than thoracotomy for administration of adjuvant chemotherapy after lobectomy for non-small cell lung cancer. World Journal of Surgical Oncology 9.

65. Jiang W, Chen X, Xi J, Wang Q (2013) Selective mediastinal lymphadenectomy without intraoperative frozen section examinations for clinical stage I non-small-cell lung cancer: Retrospective study of 403 cases. World Journal of Surgery 37: 392-397.

66. Kaseda S (2005) Video assisted thoracic surgery (VATS) lobectomy combined with lymphadenectomy as a new modality for the treatment of lung cancer. Japanese Journal of Lung Cancer 45: 255-260.

67. Kawai H, Tayasu Y, Saitoh A, Ooyama K, Tanaka Y, et al. (2005) Nocturnal hypoxemia after lobectomy for lung cancer. Ann Thorac Surg 79: 1162-1166.

68. Koizumi K, Haraguchi S, Hirata T, Hirai K, Mikami I, et al. (2003) Video-assisted lobectomy for a lung cancer patient with chronic obstructive pulmonary disease. Jpn J Thorac Cardiovasc Surg 51: 569-576.

69. Koizumi K, Haraguchi S, Hirata T, Hirai K, Mikami I, et al. (2005) Surgical treatment for lung cancer patients with poor pulmonary function. Ann Thorac Cardiovasc Surg 11: 87-92.

70. Koren JP, Bocage JP, Geis WP, Caccavale RJ (2003) Major thoracic surgery in octogenarians: The video-assisted thoracic surgery (VATS) approach. Surgical Endoscopy and Other Interventional Techniques 17: 632-635.

71. Kuritzky AM, Ryder BA, Ng T (2013) Long-term survival outcomes of Video-assisted Thoracic Surgery (VATS) lobectomy after transitioning from open lobectomy. Ann Surg Oncol 20: 2734-2740.

72. Lee JG, Cho BC, Bae MK, Lee CY, Park IK, et al. (2011) Thoracoscopic lobectomy is associated with superior compliance with adjuvant chemotherapy in lung cancer. Annals of Thoracic Surgery 91: 344-349.

73. Lee PC, Nasar A, Port JL, Paul S, Stiles B, et al. (2013) Long-term survival after lobectomy for non-small cell lung cancer by video-assisted thoracic surgery versus thoracotomy. Annals of Thoracic Surgery 96: 951-961.

74. Li FW, Jiang GC, Li Y, Bu L, Yang F, et al. (2011) [Preliminary comparison research of thoracoscopy and thoracotomy lobectomy for clinical N0 and post-operatively pathological N2 non-small cell lung cancer]. Beijing da xue xue bao Yi xue ban = Journal of Peking University Health sciences 43: 861-865.

75. Li X, Wang J, Ferguson MK (2013) Competence versus mastery: The time course for developing proficiency in video-assisted thoracoscopic lobectomy. J Thorac Cardiovasc Surg.

76. Licht PB, Jørgensen OD, Ladegaard L, Jakobsen E (2013) A national study of nodal upstaging after thoracoscopic versus open lobectomy for clinical stage I lung cancer. Annals of Thoracic Surgery 96: 943-950.

77. Linden PA, Bueno R, Colson YL, Jaklitsch MT, Lukanich J, et al. (2005) Lung resection in patients with preoperative FEV1 < 35% predicted. Chest 127: 1984-1990.

78. Louie BE, Farivar AS, Aye RW, Vallieres E (2012) Early experience with robotic lung resection results in similar operative outcomes and morbidity when compared with matched video-assisted thoracoscopic surgery cases. Ann Thorac Surg 93: 1598-1604; discussion 1604-1595.

79. Luketich JD, Meehan MA, Landreneau RJ, Christie NA, Close JM, et al. (2000) Total videothoracoscopic lobectomy versus open thoracotomy for early-stage non-small-cell lung cancer. Clinical Lung Cancer 2: 56-60.

80. Martin-Ucar AE, Nakas A, Pilling JE, West KJ, Waller DA (2005) A case-matched study of anatomical segmentectomy versus lobectomy for stage I lung cancer in high-risk patients. European Journal of Cardio-thoracic Surgery 27: 675-679.

81. Matot I, Dery E, Bulgov Y, Cohen B, Paz J, et al. (2013) Fluid management during video-assisted thoracoscopic surgery for lung resection: a randomized, controlled trial of effects on urinary output and postoperative renal function. J Thorac Cardiovasc Surg 146: 461-466.

82. Matsuoka K, Kuroda A, Kang A, Imanishi N, Nagai S, et al. (2013) Video-assisted thoracoscopic surgery for lung cancer in patients on hemodialysis. Ann Thorac Cardiovasc Surg 19: 263-267.

83. McKenna RJ, Jr., Houck W, Fuller CB (2006) Video-assisted thoracic surgery lobectomy: experience with 1,100 cases. Ann Thorac Surg 81: 421-425; discussion 425-426.

84. Meyerson SL, Gustafson MR (2011) Short-term outcomes after thoracoscopic lobectomy in elderly compared to younger patients. Innovations: Technology and Techniques in Cardiothoracic and Vascular Surgery 6: 28-31.

85. Möller A, Sartipy U (2012) Predictors of postoperative quality of life after surgery for lung cancer. Journal of Thoracic Oncology 7: 406-411.

86. Mun M, Kohno T (2008) Video-Assisted Thoracic Surgery for Clinical Stage I Lung Cancer in Octogenarians. Annals of Thoracic Surgery 85: 406-411.

87. Nakanishi R, Nakagawa M, Tokufuchi H, Okumura T, Maeda M, et al. (2012) Video-assisted thoracoscopic lobectomy for clinical stage I non-small cell lung cancer: Experience with 111 consecutive patients demonstrating comorbidity. Minerva Chirurgica 67: 67-75.

88. Nakanishi R, Yamashita T, Oka S (2010) Video-assisted thoracic surgery lobectomy for non-small cell lung cancer in patients with a Charlson comorbidity index score of two or more. J Thorac Oncol 5: 56-61.

89. Nicastri DG, Wisnivesky JP, Litle VR, Yun J, Chin C, et al. (2008) Thoracoscopic lobectomy: report on safety, discharge independence, pain, and chemotherapy tolerance. J Thorac Cardiovasc Surg 135: 642-647.

90. Nojiri T, Inoue M, Yamamoto K, Maeda H, Takeuchi Y, et al. (2011) B-type natriuretic Peptide as a predictor of postoperative cardiopulmonary complications in elderly patients undergoing pulmonary resection for lung cancer. Ann Thorac Surg 92: 1051-1055.

91. Onaitis MW, Petersen RP, Balderson SS, Toloza E, Burfeind WR, et al. (2006) Thoracoscopic lobectomy is a safe and versatile procedure: experience with 500 consecutive patients. Ann Surg 244: 420-425.

92. Papiashvilli M, Sasson L, Azzam S, Hayat H, Schreiber L, et al. (2013) Video-assisted thoracic surgery lobectomy versus lobectomy by thoracotomy for lung cancer: Pilot study. Innovations: Technology and Techniques in Cardiothoracic and Vascular Surgery 8: 6-11.

93. Papiashvilli M, Stav D, Cyjon A, Haitov Z, Gofman V, et al. (2012) Lobectomy for non-small cell lung cancer: Differences in morbidity and mortality between thoracotomy and thoracoscopy. Innovations: Technology and Techniques in Cardiothoracic and Vascular Surgery 7: 15-22.

94. Park HS, Detterbeck FC, Boffa DJ, Kim AW (2012) Impact of hospital volume of thoracoscopic lobectomy on primary lung cancer outcomes. Annals of Thoracic Surgery 93: 372-379.

95. Petersen RP, Pham D, Toloza EM, Burfeind WR, Harpole DH, Jr., et al. (2006) Thoracoscopic lobectomy: a safe and effective strategy for patients receiving induction therapy for non-small cell lung cancer. Ann Thorac Surg 82: 214-218; discussion 219.

96. Pu Q, Ma L, Mei J, Zhu Y, Che G, et al. (2013) Video-assisted thoracoscopic surgery versus posterolateral thoracotomy lobectomy: A more patient-friendly approach on postoperative pain, pulmonary function and shoulder function. Thoracic Cancer 4: 84-89.

97. Rivera C, Dahan M, Bernard A, Falcoz PE, Thomas P (2011) Surgical treatment of lung cancer in the octogenarians: results of a nationwide audit. Eur J Cardiothorac Surg 39: 981-986.

98. Rivera C, Falcoz PE, Bernard A, Thomas PA, Dahan M (2011) Surgical management and outcomes of elderly patients with early stage non-small cell lung cancer: A nested case-control study. Chest 140: 874-880.

99. Roviaro G, Varoli F, Vergani C, Nucca O, Maciocco M, et al. (2004) Long-term survival after videothoracoscopic lobectomy for stage I lung cancer. Chest 126: 725-732.

100. Saha SP, Bender M, Ferraris VA, Davenport DL (2013) Surgical treatment of lung cancer in octogenarians. Southern Medical Journal 106: 356-361.

101. Samson P, Guitron J, Reed MF, Hanseman DJ, Starnes SL (2013) Predictors of conversion to thoracotomy for video-assisted thoracoscopic lobectomy: A retrospective analysis and the influence of computed tomography-based calcification assessment. Journal of Thoracic and Cardiovascular Surgery 145: 1512-1518.

102. Scott WJ, Matteotti RS, Egleston BL, Oseni S, Flaherty JF (2010) A comparison of perioperative outcomes of Video-Assisted Thoracic Surgical (VATS) Lobectomy with open thoracotomy and lobectomy: Results of an analysis using propensity score based weighting. Annals of Surgical Innovation and Research 4.

103. Seder CW, Hanna K, Lucia V, Boura J, Kim SW, et al. (2009) The Safe Transition from Open to Thoracoscopic Lobectomy: A 5-Year Experience. Annals of Thoracic Surgery 88: 216-226.

104. Shaw JP, Dembitzer FR, Wisnivesky JP, Litle VR, Weiser TS, et al. (2008) Video-assisted thoracoscopic lobectomy: state of the art and future directions. Ann Thorac Surg 85: S705-709.

105. Shiraishi T, Shirakusa T, Miyoshi T, Hiratsuka M, Yamamoto S, et al. (2006) A completely thoracoscopic lobectomy/segmentectomy for primary lung cancer--technique, feasibility, and advantages. Thorac Cardiovasc Surg 54: 202-207.

106. Swanson SJ, Miller DL, McKenna RJ, Jr., Howington J, Marshall MB, et al. (2013) Comparing robot-assisted thoracic surgical lobectomy with conventional video-assisted thoracic surgical lobectomy and wedge resection: Results from a multihospital database (Premier). J Thorac Cardiovasc Surg.

107. Tajiri M, Maehara T, Nakayama H, Sakamoto K (2007) Decreased invasiveness via two methods of thoracoscopic lobectomy for lung cancer, compared with open thoracotomy. Respirology 12: 207-211.

108. Tovar EA (2001) One-day admission for major lung resections in septuagenarians and octogenarians: A comparative study with a younger cohort. European Journal of Cardio-thoracic Surgery 20: 449-454.

109. Tsunezuka Y, Waseda R, Yachi T (2010) Electrothermal bipolar vessel sealing device LigaSureV for pulmonary artery ligation--burst pressure and clinical experiences in complete video-assisted thoracoscopic major lung resection for lung cancer. Interact Cardiovasc Thorac Surg 11: 229-233.

110. Ueda K, Hayashi M, Tanaka T, Hamano K (2013) Omitting chest tube drainage after thoracoscopic major lung resection. European Journal of Cardio-thoracic Surgery 44: 225-229.

111. Ueda K, Sudoh M, Jinbo M, Li TS, Suga K, et al. (2006) Physiological rehabilitation after video-assisted lung lobectomy for cancer: a prospective study of measuring daily exercise and oxygenation capacity. Eur J Cardiothorac Surg 30: 533-537.

112. Van't Westeinde SC, Horeweg N, De Leyn P, Groen HJ, Lammers JW, et al. (2012) Complications following lung surgery in the Dutch-Belgian randomized lung cancer screening trial. Eur J Cardiothorac Surg 42: 420-429.

113. Verstegen NE, Oosterhuis JWA, Palma DA, Rodrigues G, Lagerwaard FJ, et al. (2013) Stage I-II non-small-cell lung cancer treated using either stereotactic ablative radiotherapy (SABR) or lobectomy by video-assisted thoracoscopic surgery(VATS): Outcomes of a propensity score-matchedanalysis. Annals of Oncology 24: 1543-1548.

114. Villamazar N (2009) Thoracoscopic lobectomy is associated with lower morbidity compared with thoracotomy. Annals of Thoracic Surgery.

115. Walker WS, Codispoti M, Soon SY, Stamenkovic S, Carnochan F, et al. (2003) Long-term outcomes following VATS lobectomy for non-small cell bronchogenic carcinoma. Eur J Cardiothorac Surg 23: 397-402.

116. Wan IY, Thung KH, Hsin MK, Underwood MJ, Yim AP (2008) Video-assisted thoracic surgery major lung resection can be safely taught to trainees. Ann Thorac Surg 85: 416-419.

117. Watanabe A, Osawa H, Watanabe T, Mawatari T, Ichimiya Y, et al. (2003) Complications of major lung resections by video-assisted thoracoscopic surgery. Kyobu geka The Japanese journal of thoracic surgery 56: 943-948.

118. Whitson BA, Andrade RS, Boettcher A, Bardales R, Kratzke RA, et al. (2007) Video-Assisted Thoracoscopic Surgery is More Favorable Than Thoracotomy for Resection of Clinical Stage I Non-Small Cell Lung Cancer. Annals of Thoracic Surgery 83: 1965-1970.

119. Yamashita Y, Mukaida H, Harada H, Tsubokawa N (2013) Post-thoracotomy pain and long-term survival associated with video-assisted thoracic surgery lobectomy methods for clinical T1N0 lung cancer: A patient-oriented, prospective cohort study. European Journal of Cardio-thoracic Surgery 44: e71-e76.

120. Yellin A, Simansky D, Paley M, Refaely Y (2002) Video-assisted thoracoscopic lobectomy--preliminary experience. Harefuah 141: 138-141, 224.

121. Yim AP, Wan S, Lee TW, Arifi AA (2000) VATS lobectomy reduces cytokine responses compared with conventional surgery. Ann Thorac Surg 70: 243-247.

122. Zhang L, Ma W, Li Y, Jiang Y, Ma G, et al. (2013) Comparative study of the anatomic segmentectomy versus lobectomy for clinical stage IA peripheral lung cancer by video assistant thoracoscopic surgery. Journal of Cancer Research and Therapeutics 9: S106-S109.

123. Zhang Y, Jiang Gn, Gao W, Chen C (2012) Impact of compromised pulmonary function on major lung resection for non-small cell lung cancer: Retrospective study of 127 cases. Chinese Medical Journal 125: 3465-3471.

124. Zhang Y, Jiang GN, Wang Q, Zhu YM, Ding JA, et al. (2010) [Cytokine responses after lobectomy for early non-small cell lung cancer: a prospective randomized comparison of video-assisted thoracic surgery and open thoracotomy]. Zhonghua Wai Ke Za Zhi 48: 1285-1288.

125. Zhu J, Zhao TJ, Chen HZ, Yang LX, Jin H, et al. (2013) Surgical procedure and prognosis analysis for elderly patients with non-small-cell lung carcinoma. Academic Journal of Second Military Medical University 34: 1021-1024.

126. Endoh H, Tanaka S, Yajima T, Ito T, Tajima K, et al. (2010) Pulmonary function after pulmonary resection by posterior thoracotomy, anterior thoracotomy or video-assisted surgery. European Journal of Cardio-thoracic Surgery 37: 1209-1214.

127. Nagahiro I, Andou A, Aoe M, Sano Y, Date H, et al. (2001) Pulmonary function, postoperative pain, and serum cytokine level after lobectomy: A comparison of VATS and conventional procedure. Annals of Thoracic Surgery 72: 362-365.

128. Nakamura H, Taniguchi Y, Miwa K, Adachi Y, Fujioka S, et al. (2011) Comparison of the surgical outcomes of thoracoscopic lobectomy, segmentectomy, and wedge resection for clinical stage i non-small cell lung cancer. Thoracic and Cardiovascular Surgeon 59: 137-141.

129. Nakata M, Saeki H, Yokoyama N, Kurita A, Takiyama W, et al. (2000) Pulmonary function after lobectomy: Video-assisted thoracic surgery versus thoracotomy. Annals of Thoracic Surgery 70: 938-941.

130. Okada S, Sugawara H, Tanaba Y, Ishimori S, Yamauchi T, et al. (2000) [Thoracoscopic major lung resection using a newly developed instrument retraction system and a voice-controlled robot]. Kyobu Geka 53: 862-865.

131. Whitson BA, D'Cunha J, Andrade RS, Kelly RF, Groth SS, et al. (2008) Thoracoscopic versus thoracotomy approaches to lobectomy: differential impairment of cellular immunity. Ann Thorac Surg 86: 1735-1744.

132. Burfeind WR, D'Amico TA (2004) Thoracoscopic lobectomy. Operative Techniques in Thoracic and Cardiovascular Surgery 9: 98-114.

133. Hartwig MG, D'Amico TA (2010) Thoracoscopic Lobectomy: The Gold Standard for Early-Stage Lung Cancer? Annals of Thoracic Surgery 89: S2098-S2101.

134. Mehta HJ, Ross C, Silvestri GA, Decker RH (2011) Evaluation and Treatment of High-Risk Patients with Early-Stage Lung Cancer. Clinics in Chest Medicine 32: 783-797.
